# Serum Vitamin B12 Levels as a Risk Factor and Prognostic Marker in Patients With Acute Ischemic Stroke at a Tertiary Care Center in Northern India: A Case-Control Study

**DOI:** 10.7759/cureus.70473

**Published:** 2024-09-29

**Authors:** Virendra Atam, Sagar Srivastava, Akashdeep Sharma, Isha Atam, Jay Tewari, Khalid A Qidwai

**Affiliations:** 1 Internal Medicine, King George's Medical University, Lucknow, IND; 2 Internal Medicine, Gandhi Memorial and Associated Hospitals, King George's Medical University, Lucknow, IND

**Keywords:** acute ischemic stroke, prognosis, risk factor, stroke, vitamin b12

## Abstract

Objective

Stroke is a leading cause of death and disability globally, with its incidence, prevalence, and mortality rising significantly over the past decades. Beyond traditional risk factors, vitamin B12 has garnered attention for its role in stroke prevention due to its influence on homocysteine metabolism. Deficiencies in vitamin B12 are linked to hyperhomocysteinemia, which increases the risk of ischemic stroke. This study aims to compare vitamin B12 and homocysteine levels in stroke patients versus control subjects.

Methodology

This observational case-control study was conducted at King George’s Medical University (KGMU), Lucknow, involving 75 acute ischemic stroke patients and 75 age- and sex-matched controls. Serum vitamin B12 and homocysteine levels were measured, and stroke severity was assessed using the National Institutes of Health Stroke Scale (NIHSS) score. The modified Rankin scale (MRS) evaluated functional outcomes at discharge. Statistical analysis was performed to identify associations between vitamin B12 levels, stroke severity, and patient outcomes.

Results

Stroke patients had significantly lower vitamin B12 levels (194.24 ± 91.11 pmol/L) and higher homocysteine levels (16.33 ± 3.29 µmol/L) compared to controls (271.13 ± 91.19 pmol/L and 9.76 ± 4.55 µmol/L, respectively). Vitamin B12 levels were lower, and homocysteine levels were higher in patients who died during the study. Additionally, vitamin B12 levels were negatively correlated with MRS scores at discharge and 28 days post-discharge, and positively correlated with NIHSS scores, indicating worse outcomes in patients with lower vitamin B12 levels.

Conclusions

This study demonstrates a significant association between vitamin B12 deficiency and the occurrence and severity of ischemic stroke. Lower vitamin B12 levels correlated with higher stroke severity and poorer functional outcomes, highlighting the potential role of vitamin B12 in stroke management. Further research is needed to explore the therapeutic implications of vitamin B12 supplementation in reducing stroke risk and improving patient outcomes.

## Introduction

Stroke ranks second among the causes of death in India and worldwide. It is the third biggest cause of death and disability combined. In 2019, the incidence, prevalence, and mortality due to stroke were 12·2 million (an increase of 70% since 1990), 101 million (an increase of 85% since 1990), and 6·55 million (an increase of 43% since 1990), respectively [1].

The World Health Organization (WHO) has defined stroke as “rapidly developed clinical signs of focal (or global) disturbance of cerebral function, lasting more than 24 hours or leading to death, with no apparent cause other than of vascular origin” [2].

Beyond traditional risk factors, nutrients like vitamin B12 are gaining attention in ischemic stroke prevention. Vitamin B12 is essential for DNA synthesis, blood cell production, and nerve function. It may protect against stroke through antioxidant properties, mitochondrial regulation, immune modulation, and antiatherosclerosis. Low vitamin B12 levels are linked to higher stroke risk and worse outcomes [3].

Vitamin B12 helps convert homocysteine (Hcy) to methionine, which also involves the presence of folic acid. Deficiencies in Vitamin B12 or folate thus cause hyperhomocysteinemia, increasing ischemic stroke risk as Hcy is associated with an increased risk of thrombosis by its various mechanisms of action [4]. Studies show high plasma Hcy levels are associated with small vessel stroke. Supplementing folic acid and B12 may reduce Hcy levels and lower stroke risk [3].

Vitamin B12 deficiency, defined as serum vitamin B12 <200 pg/mL (148 pmol/L), affects about 3.6% of all adults aged 19 years and older, and 3.7% of those aged 60 years and above, according to an analysis of National Health and Nutrition Examination Survey (NHANES) data from 2007 to 2018. On the other hand, vitamin B12 insufficiency is more common, impacting about 12.5% of all adults aged 19 and older and 12.3% of those aged 60 and older (defined as serum vitamin B12 <300 pg/mL [221 pmol/L]) [5].

However, few prospective studies have examined the links between folate or vitamin B12 levels and the risk of stroke, and the results have been inconsistent. Therefore, this study aims to compare vitamin B12 and Hcy levels in stroke patients with those in control subjects.

## Materials and methods

The study aimed to measure serum vitamin B12 levels in patients with acute ischemic stroke and compare these levels with age and sex-matched controls. Additionally, it sought to analyze stroke severity using the NIHSS score and evaluate patient functional outcomes at discharge based on the modified Rankin scale (MRS). This study was approved by the Institutional Ethics Committee of King George's Medical University (KGMU), UP (reference code: XVII-PGTSC-IIA/P19). Conducted in the Department of Medicine at KGMU in Lucknow, the study spanned one year and followed an observational case-control design. The sample size comprised 75 acute ischemic stroke cases and 75 controls.

Participants were included if they were over 12 years old, consented to participate, and met the WHO definition of acute stroke confirmed by radiological investigations. Controls were age and sex-matched individuals without stroke from the Medical OPD. Exclusion criteria included patients with hemorrhagic stroke, transient ischemic attacks, inconsistent clinical and radiological stroke findings, head injuries, intracranial space-occupying lesions, chronic kidney disease, or folic acid levels ≥ 3 ng/ml.

A total of 150 participants (75 cases and 75 controls) were enrolled after giving informed consent. Each participant underwent a thorough evaluation, including a detailed history, clinical examination, and severity assessment using the NIHSS score at admission. Stroke severity was categorized as minor (NIHSS = 1-4), moderate (NIHSS = 5-15), moderate to severe (NIHSS = 16-20), and severe (NIHSS = 21-42). Clinical history focused on stroke symptoms and risk factors such as smoking, alcohol consumption, diabetes, hypertension, dyslipidemia, coronary artery disease, and rheumatic heart disease. Neurological deficits were identified through detailed clinical examination.

The study involved extensive investigations including complete blood counts, random blood glucose, serum electrolytes, kidney and liver function tests, HbA1C, fasting lipid profile, ECG, prothrombin time, INR, routine urine analysis, echocardiography, chest X-ray, CT and MRI scans of the brain, serum vitamin B12 levels, and Hcy levels. Serum vitamin B12 levels and serum Hcy were measured using a chemiluminescent immunoassay, and participants were categorized based on their levels into normal, metabolic deficiency, and biochemical deficiency groups.

All blood investigations followed standard protocols and were carried out in KGMU’s pathology laboratory and radiology department. During the hospital stay, patients were monitored for length of stay and outcomes at discharge. Disability at admission and discharge was assessed using the MRS, with scores ≤ 2 indicating a good outcome and scores >2 indicating a poor outcome.

Data were recorded in a Master Chart and analyzed using SPSS software version 29.0 (IBM Corp., Armonk, NY). Results were presented as mean ± standard deviation of the mean. Statistical analysis included T-tests, F-tests, Pearson correlation coefficient calculations, and Chi-square tests, with *P*-values < 0.05 considered statistically significant.

## Results

In this study, age and sex-matched a total of 75 cases (acute ischemic stroke) and 75 controls were enrolled. The baseline characteristics of the cases and the controls have been shown in Table 1. The cases and controls had a matched distribution of anti-diabetic agents used; among the 33 diabetic cases, 32 were on metformin and one was on insulin. Among the 29 diabetic controls, 27 were on metformin, one was on no medication, and one was on insulin.

**Table 1:** Baseline characteristics of cases and controls. Data are presented either in the form of *n *(%), number of individuals (percentage) or mean ± standard deviation.

Characteristics	Cases (n = 75)	Controls (*n* = 75)
Age	52.69 ± 13.11	53.26 ± 14.61
Males	41 (55%)	39 (52%)
Females	34 (45%)	36 (48%)
Hypertension	40 (53.3%)	38 (50.6%)
Type 2 diabetes mellitus	33 (44%)	29 (38.6%)
Dyslipidemia	39 (52%)	35 (46.6%)

Table 2 shows the mean serum levels of vitamin B12 and serum Hcy in the cases and the controls. Vitamin B12 was significantly decreased and Hcy was significantly increased in the case group.

**Table 2:** Association of mean vitamin B12 (pmol/L) with case and control. Data are presented in the form of mean ± standard deviation.

Biomarkers	Cases (*n* = 75)	Controls (*n* = 75)	Chi^2^	*P*-value
Vitamin B12 (pmol/L/mL)	194.24 ± 91.11	271.13 ± 91.19	-5.17	<0.001
Serum homocysteine level (µmol/L)	16.33 ± 3.29	9.76 ± 4.55	10.14	<0.001

Table 3 shows the association of frequencies of normal, biochemical deficiency, and metabolic deficiency between cases and controls. 

**Table 3:** Association of frequencies of normal, biochemical, and metabolic with case and control. Data are presented either in the form of *n* (%), number of individuals (percentage).

Vitamin B12 Level	Cases (*n *= 75)	Controls (*n *= 75)	Chi^2^	*P*-value
Normal	10 (13.33%)	55 (73.33%)	56.14	<0.001
Biochemical deficiency	28 (37.33%)	12 (16%)		
Metabolic deficiency	37 (49.33%)	8 (10.67%)		

Table 4 shows the association of frequencies of different stroke types (Trial of Org 10172 in Acute Stroke Treatment [TOAST] classification), serum vitamin B12, and serum Hcy levels with survival status. The mean vitamin B12 (pmol/L) was 214.84 ± 93.13 in the living group and 134.05 ± 47.71 in the death group. In addition, the vitamin B12 (pmol/L) was significantly lower in the death group as compared to live patients. The biochemical deficiency of vitamin B12 was significantly higher in the patients in the death group than in the living group. The mean serum Hcy level (µmol/l) was 15.72 ± 3.43 in the living group and 18.13 ± 2.00 in the death group. In addition, the serum Hcy level (µmol/l) was significantly higher in the death group as compared to alive patients.

**Table 4:** TOAST classification subtypes; serum vitamin B12 and serum homocysteine levels and their association with survival. Data are presented either in the form of *n* (%), number of individuals (percentage) or mean ± standard deviation. TOAST, Trial of Org 10172 in Acute Stroke Treatment

Stroke type and biomarker levels		Alive (*n *= 56)	Death (*n *= 19)	Chi^2^	*P*-value
Small-vessel occlusion		26 (46.43%)	12 (63.16%)	0.83	0.660
Large-artery atherosclerosis		17 (30.36%)	5 (26.32%)		
Cardioembolic		3 (5.36%)	2 (10.53%)		
Vitamin B12 level (pmol/L)		214.84 ± 93.13	134.05 ± 47.71	-0.81	0.423
Normal vitamin B12 levels		10 (17.86%)	0 (0%)	11.61	0.003
Biochemical deficiency of vitamin B12 level		15 (26.79%)	13 (68.42%)		
Metabolic deficiency of vitamin B12 level		31 (55.36%)	6 (31.58%)		
Serum homocysteine level (µmol/L)		15.72 ± 3.43	18.13 ± 2.00	-2.41	0.019

Table 5 shows the Pearson correlation of vitamin B12 with the MRS at discharge and 28th day of discharge at discharge. Vitamin B12 was significantly negatively correlated with the MRS in ischemic stroke at discharge and 28th day of discharge.

**Table 5:** Pearson correlation of vitamin B12 with the modified Rankin scale at discharge and 28th-day post-discharge.

Variables	At discharge		28th-day post-discharge	
	Pearson correlation	*P*-value	Pearson correlation	*P*-value
Vitamin B12 and mRS score	-0.720	<0.001	-0.658	<0.001

Table 6 shows the Pearson correlation of vitamin B12 with the NIHSS severity score. Vitamin B12 was significantly positively correlated with NIHSS severity in ischemic stroke.

**Table 6:** Pearson correlation of vitamin B12 with NIHSS severity score. NIHSS, National Institutes of Health Stroke Scale

Variables	Pearson correlation	*P*-value
Vitamin B12 and NIHSS severity score	-0.720	<0.001

Table 7 shows the association of vitamin B12 with different severities of strokes (mild stroke, moderate to severe stroke, and severe stroke). The average serum vitamin B12 levels decreased with increasing severity of stroke.

**Table 7:** Association of vitamin B12 with different severity of strokes (mild stroke, moderate to severe stroke, and severe stroke). Data are presented either in the form of mean ± standard deviation. Analysis was done using the *F*-test to compare the variance in patient outcomes between the different groups to the variance within each group.

Severity of strokes	Vitamin B12	Significance
Minor stroke	277.00 ± 141.48	*P *= 0.05; *F* = 2.758
Moderate stroke	238.83 ± 105.04	
Moderate to severe stroke	191.67 ± 19.14	
Severe stroke	173.00 ± 51.71	

Table 8 shows the association of vitamin B12 with different strokes based on the TOAST classification (large artery, small artery, and cardioembolic). Participants with TOAST types 4 (stroke of other determined etiology) and 5 (stroke of undetermined etiology) were excluded from the study to ensure a more homogeneous sample focused on TOAST types 1-3. The average vitamin B12 values did not differ significantly between the different TOAST classifications.

**Table 8:** Association of vitamin B12 with different strokes based on TOAST classification (large artery, small artery, and cardioembolic). Data are presented either in the form of *n *(%), number of individuals (percentage) or mean ± standard deviation. The analysis was done using the *F*-test to compare the variance in patient outcomes between the different groups to the variance within each group. TOAST, Trial of Org 10172 in Acute Stroke Treatment

TOAST classification	*n* (%)	Vitamin B12	Significance
Small-vessel occlusion	36 (64.3%)	220.17 ± 106.86	*P *= 0.84; *P *= 0.17
Large-artery atherosclerosis	17 (30.35%)	206.41 ± 64.87	
Cardioembolic	3 (5.35%)	198.67 ± 59.14	

## Discussion

Our case-control study evaluated the potential role of serum vitamin B12 levels as a risk factor and a prognostic marker for acute ischemic stroke between 75 cases and 75 controls, which were age and sex-matched; other potential traditional risk factors were matched like hypertension, diabetes mellitus, and dyslipidemia. Cases were found to have significantly lower vitamin B12 levels (194.24 ± 91.11 pmol/L) compared to controls (271.13 ± 91.19 pmol/L). Serum Hcy levels were higher in cases (16.33 ± 3.29 µmol/L) in comparison to the controls (9.76 ± 4.55 µmol/L). In comparing the stroke types, small-vessel occlusion strokes were higher in the death group (63.16%) than in the living group (46.43%). Vitamin B12 levels were lower in the death group (134.05 ± 47.71 pmol/L) compared to the living group (214.84 ± 93.13 pmol/L). Hcy levels were higher in the death group (18.13 ± 2.00 µmol/L) compared to the living group (15.72 ± 3.43 µmol/L). Vitamin B12 levels negatively correlated with the MRS at discharge and 28 days post-discharge and positively correlated with NIHSS severity scores. The mean vitamin B12 levels did not significantly differ between different TOAST classification stroke types.

A lack of vitamin B12 causes the body to produce higher levels of Hcy because it is a cofactor involved in the methylation of Hcy to methionine. Hcy has been identified as a pro-thrombotic molecule that functions through a variety of pathways, including increased expression of tissue factors, inhibition of anticoagulant processes, augmentation of platelet reactivity, thrombin generation and factor V activity, impairment of fibrinolysis and vascular endothelial function. Increased oxidative stress, DNA hypomethylation, and proinflammatory effects of Hcy are implicated as the molecular mechanisms underlying its prothrombotic actions [4].

Both methylmalonic acid and Hcy levels rise in vitamin B12 deficiency. While methylmalonic acid measurement requires mass spectrometry, Hcy can be easily measured via various clinical assays. Hcy levels strongly correlate with methylmalonic acid, with over 95% sensitivity in detecting vitamin B12 deficiency [6-8]. Therefore, serum Hcy levels can be measured as a proxy for vitamin B12 levels.

In a recent study by Liang et al., elevated Hcy levels were implicated in increased all-cause mortality and mortality due to cardiovascular diseases [9]. Conversely, the conclusion can be drawn that decreased vitamin B12 levels are associated with worse prognosis in people with cardiovascular disease. The association between serum vitamin B complex deficiencies and the risk of stroke has been mentioned in the medical literature often but the strength and direction of the association have been inconsistent across the studies [3]. In a 15-year follow-up study involving 43,732 men between the ages of 40 and 75, He et al. reported a 30% reduction in the risk of ischemic stroke with folic acid and vitamin B12 supplementation [10]. The meta-analysis by Jenkins et al., which evaluated the role of supplemental vitamins and minerals for the prevention and treatment of cardiovascular diseases revealed that folate and vitamin B12 supplementation reduced the risk of stroke [11]. A Mendelian randomization study by Yuan et al. found that levels of folate and vitamin B6 had a genetic disposition for ischemic strokes but vitamin B12 failed to show this effect [12]. The study by Lonn et al. showed no significant benefit from vitamin B supplementation on cardiovascular outcomes. This study included 5,522 patients over 55 years of age [13].

The TOAST classification method categorizes ischemic strokes into etiological categories [14]. Our study found no differences in vitamin B12 levels between these subcategories, suggesting other factors may primarily contribute to stroke development.

Mean vitamin B12 values were lower in severe and moderate to severe strokes compared to minor and moderate strokes, indicating a significant decrease with increasing stroke severity. Stroke severity is influenced by multiple factors, including stroke size and location, and comorbidities like hypertension, diabetes, and atrial fibrillation. Thus, the relationship between vitamin B12 levels and NIHSS scores may be confounded by other variables, necessitating further research. In our study, normal, biochemical, and metabolic vitamin B12 deficiency percentages were 17.86%, 26.79%, and 55.36% in the living group, and 0.00%, 68.42%, and 31.58% in the death group, respectively, which was statistically significant. Biochemical vitamin B12 deficiency was significantly higher in the death group than in the living group.

This case-control study provides evidence justifying the role of Vitamin B12 deficiency in the development and prognosis of acute ischemic stroke. Serum vitamin B12 levels can be measured easily and on a routine basis to screen people for potential risk of cardiovascular diseases, especially stroke. This study took place in a tertiary care center in Northern India hence it also provides an epidemiological view of the biochemical profile of people with stroke. The cases and controls were thoroughly matched across age, sex, and potential traditional risk factors for stroke which further strengthens our claims.

Limitations of the study

Our study had some limitations. The sample size was relatively small, and since the study was conducted in northern India, it may not represent all ethnicities across the country. This calls for more high-quality observational studies and randomized controlled trials to be conducted across various geographical regions.

## Conclusions

This case-control study underscores a significant relationship between the occurrence and severity of ischemic stroke and serum vitamin B12 deficiency. Stroke patients had notably lower vitamin B12 levels and higher serum Hcy levels compared to controls, highlighting the potential role of vitamin B12 deficiency in stroke pathology. The study also found that patients with lower vitamin B12 and higher Hcy levels had poorer outcomes, including increased mortality. 

Furthermore, a negative correlation between vitamin B12 levels and MRS scores at discharge and 28 days post-discharge suggests that higher vitamin B12 levels are associated with better functional outcomes. The positive correlation between vitamin B12 deficiency and NIHSS severity scores reinforces the impact of vitamin B12 on stroke severity. Overall, the findings emphasize the importance of maintaining adequate vitamin B12 levels in managing ischemic stroke, suggesting that addressing vitamin B12 deficiency may help reduce stroke severity and improve patient outcomes.
